# Medication review plus person-centred care: a feasibility study of a pharmacy-health psychology dual intervention to improve care for people living with dementia

**DOI:** 10.1186/s12888-018-1907-4

**Published:** 2018-10-19

**Authors:** Ian D Maidment, Sarah Damery, Niyah Campbell, Nichola Seare, Chris Fox, Steve Iliffe, Andrea Hilton, Graeme Brown, Nigel Barnes, Jane Wilcock, Emma Randle, Sarah Gillespie, Garry Barton, Rachel Shaw

**Affiliations:** 1 School of Life and Health Sciences, Aston University, Birmingham, B4 7ET UK; 2 Institute of Applied Health Research, College of Medical and Dental Sciences, University of Birmingham, Edgbaston, Birmingham, B15 2TT UK; 3 Aston Health Research Innovation Cluster, Aston University, Birmingham, B4 7ET UK; 4 Norwich Medical School, University of East Anglia, Earlham Road, Norwich, Norfolk NR4 7TJ UK; 5 Research Department of Primary Care & Population Health, University College London, Royal Free Campus, Rowland Hill St, London, NW3 2PF UK; 6 Faculty of Health Science, University of Hull, Hull, HU6 7RX UK; 7 Birmingham and Solihull Mental Health NHS Foundation Trust, Unit 1, B1, 50 Summer Hill Road, Birmingham, B1 3RB UK; 8 National Centre for Mental Health, Research and Innovation Department, The Barberry, 25 Vincent Drive, Birmingham, B15 2FG UK; 9 Department of Clinical Healthcare, Faculty of Health and Life Sciences, Oxford Brookes University, Gipsy Lane Campus, Headington, Oxford, OX3 0FL UK; 10 Norwich Clinical Trials Unit, University of East Anglia, Earlham Road, Norwich, Norfolk NR4 7TJ UK

**Keywords:** Dementia, Primary care, Care homes, Pharmacy, Medication review, Behaviour that challenges

## Abstract

**Background:**

“Behaviour that Challenges” is common in people living with dementia, resident in care homes and historically has been treated with anti-psychotics. However, such usage is associated with 1800 potentially avoidable deaths annually in the UK. This study investigated the feasibility of a full clinical trial of a specialist dementia care pharmacist medication review combined with a health psychology intervention for care staff to limit the use of psychotropics.

This paper focuses on feasibility; including recruitment and retention, implementation of medication change recommendations and the experiences and expectations of care staff.

**Methods:**

West Midlands care homes and individuals meeting the inclusion criteria (dementia diagnosis; medication for behaviour that challenges), or their personal consultee, were approached for consent.

A specialist pharmacist reviewed medication. Care home staff received an educational behaviour change intervention in a three-hour session promoting person-centred care. Primary healthcare staff received a modified version of the training.

The primary outcome measure was the Neuropsychiatric Inventory-Nursing Home version at 3 months. Other outcomes included quality of life, cognition, health economics and prescribed medication. A qualitative evaluation explored expectations and experiences of care staff.

**Results:**

Five care homes and 34 of 108 eligible residents (31.5%) were recruited, against an original target of 45 residents across 6 care homes. Medication reviews were conducted for 29 study participants (85.3%) and the pharmacist recommended stopping or reviewing medication in 21 cases (72.4%). Of the recommendations made, 57.1% (12 of 21) were implemented, and implementation (discontinuation) took a mean of 98.4 days. In total, 164 care staff received training and 21 were interviewed.

Care staff reported a positive experience of the intervention and post intervention adopting a more holistic patient-centred approach.

**Conclusions:**

The intervention contained two elements; staff training and medication review. It was feasible to implement the staff training, and the training appeared to increase the ability and confidence of care staff to manage behaviour that challenges without the need for medication. The medication review would require significant modification for full trial partly related to the relatively limited uptake of the recommendations made, and delay in implementation.

**Trial registration:**

ISRCTN58330068. Registered 15 October 2017. Retrospectively registered

## Background

Dementia is an international healthcare priority [1, 2]. One of the key challenges within dementia care is the management of the behavioural and psychological symptoms of dementia (BPSD) [3]. Behavioural symptoms include aggression, agitation, depression and hallucinations [4]. BPSD is also referred to as behaviour that challenges, which is defined as ‘any behaviour considered antisocial within the care environment or deemed dangerous to the person with dementia, their fellow residents, and staff’ [5]. These two terms are used interchangeably in this paper.

Antipsychotics are frequently prescribed for people living with dementia for behaviour that challenges [3]. The usage of antipsychotics for people with BPSD is implicated in the death of 1800 people every year and two-thirds of such usage may be inappropriate [4]. The Banerjee Report found antipsychotics were often used as a first solution, yet behaviour that challenges can often be safely managed with the use of non-pharmacological approaches [2, 4]. Senior staff in care homes should be skilled in appropriate non-pharmacological techniques and be able to train other staff in these techniques [2, 4].

A recent Cochrane review concluded that antipsychotics could be successfully discontinued in older people with dementia and BPSD, but the evidence was low quality and further research was required [6]. Furthermore, solely focussing on the prescription of antipsychotics may simply drive prescribing to equally problematic alternative psychotropics (such as anti-depressants and benzodiazepines) and research should test interventions to limit the use of all psychotropics [6, 7]. Secondary care specialist dementia care pharmacists could have a vital role in ensuring the appropriate use of psychotropics for BPSD [4, 8].

This feasibility study was designed to provide key information on study processes and outcomes, so that the challenges in implementing and evaluating a pharmacy-health psychology dual intervention could be understood [9]. Incorporating learning from a feasibility study can enhance the rigour and deliverability of any subsequent full clinical trial [10, 11]. A feasibility study was needed to assess implementation of the protocol and estimate key parameters, such as recruitment, consent and follow-up rates, and the time taken to conduct the study, to inform the design of the main trial [9, 12]. It was also conducted to refine the battery of outcome measures, and understand any challenges associated with joint working between care homes, general practitioners (GPs) and pharmacists.

### Aim

To determine whether it is feasible to implement and measure the effectiveness of a dual purpose pharmacy–health psychology intervention incorporating medication review and staff training to limit the prescription of psychotropics to manage BPSD in care home residents.

## Method

### Study design

An open label (non-blinded), mixed methods feasibility study, set within the Medical Research Council (MRC) framework for developing a complex intervention, aimed to recruit six care homes and 45 residents [13]. The study received ethical approval from National Research Ethics Services (15/EM/0314); specifically the Nottingham 1 Committee. For detailed methods refer to the published study protocol [7].

### Setting

Care homes in the West Midlands, UK. Study conducted from January 2015 until December 2017.

### Study participants

Residents in care homes recruited were eligible if inclusion criteria were met (see Table 1 for full details).

**Table 1:** Participant Inclusion and Exclusion Criteria

Participant Inclusion Criteria	
1. Receiving medication (including but not limited to medicines in British National Formulary [BNF 68] sections 4.1/4.2/4.3/4.11 to treat behaviour that challenges.	
2. Resident within a long-term care facility.	
3. Registered with a West Midlands GP (who has also agreed to participate).	
4. Dementia confirmed (dementia register, documentation of relevant read codes, confirmation of diagnosis via communication from old age psychiatry, memory clinic or clinical psychologist).	
5. Patient, or personal consultee, willing to provide consent/assent.	
6. A proxy informant (key worker or staff member with close working relationship) who can clearly communicate in English available.	
Participant Exclusion Criteria	
1. Patient, or personal consultee unable or unwilling to provide consent or lacks necessary English-language skills.	
2. On palliative care register, or has pathology requiring complex specialist medication.	
3. Risk of harm in line with Alzheimer's Society guidance (guidelines published in 2011, currently being updated and therefore not available).	
4. Severe Mental Illness (e.g. schizophrenia) where psychotropic treatment should be continued.	

### Study procedures

#### Identification and recruitment of care homes

The sampling frame was care homes (both nursing and residential) in the West Midlands (within 6 miles of Birmingham) with at least 40 residents and providing care for people living with dementia. Care Homes meeting the inclusion criteria were identified from the Care Quality Commission (CQC) and other web-sites e.g. Carehomes.co.uk including local authority sites. Eligible care homes supporting the Enabling Research in Care Homes (ENRICH; http://enrich.nihr.ac.uk/) initiative were also identified. ENRICH is an NIHR toolkit to support research in care homes. All identified care homes were invited to participate by letter with follow-up by single phone call or letter to care home manager.

#### Recruitment of residents

Consent for residents meeting the inclusion criteria was obtained from the resident, or their personal consultee, someone caring for them or interested in their welfare, but not acting in a professional capacity or for remuneration. Capacity was assessed using the Mental Capacity Act (2005) and local guidelines. All practical steps to maximise the individual’s capacity to provide informed consent, including taking sufficient time and the use of appropriate language, were taken. If the resident lacked capacity their personal consultee was approached regarding consent to the medication review (see protocol for full description of consent; [7]). The resident’s GP was then approached to consent to the medication review.

#### Recruitment of care staff

The care home manager allocated care staff to educational training sessions according to their shift patterns. Care home managers and care staff in each care home, and GPs who were involved in the medication review were invited to participate in qualitative interviews for the process evaluation.

### Intervention

The intervention contained two elements (see Table 2 for summary of the content of both elements).

**Table 2:** Description of dual-focused medication review-behavioural change intervention

Behaviour Change Intervention Overview	
“Inside Out” – An interactive face-to-face three-hour educational person-centred care group based workshop, repeated twice at each home, for care staff facilitated by a researcher with health psychology training and experience of working in the social care sector. The main aim of the intervention was to provide staff with the knowledge to: • Understand that behaviours that challenge may be an expression of unmet need Within this the intervention aimed to provide care home staff with the skills and resources to: • Investigate what the unmet need might be • Get to know the person with dementia as an individual to help manage their behaviour • Think creatively about how to prevent challenging behaviours by making sure individuals’ needs are met • Understand that behaviours that challenge are not ‘bad behaviour’ and ‘bad behaviour’ does not equate to ‘a bad person’	
Training consisted of:	
1. Educational elements about “behaviour that challenges”, the use of antipsychotics to manage behaviour that challenges, good practice guidelines to reduce psychotropics in favour of non-pharmacological interventions.	
2. Person-centred care training using the VIPS Model (V=Valuing personhood; I=Individual needs; P=Personal perspectives; S=Social environment), videos illustrating person-centred care practice and practical exercises [39].	
3. Information and discussion points emphasising the importance of self-care and good communication among care staff Primary healthcare staff received modified training primarily focussing on the treatment of BPSD.	
Summary of Medication Review (based on type 3 full clinical review)	
Medication reviews were conducted by two experienced clinical pharmacists (one who is a specialist dementia care pharmacist and has significant experience in the clinical area) and one, who acted as back-up and also has specialist experience in this area.	
1. The primary focus is to review psychotropics used to treat behaviours that challenge; the pharmacist will also review all other medication as per routine care.	
2. Establish therapeutic alliance with the person living with dementia and/or their personal consultee.	
3. Collect information from clinical records, care staff, GP and any personal consultee about the patient including prescribed treatment of BPSD, medication used to treat psychotropic induced adverse events and any other medication.	
4. Review medication, focussing on treatments for BPSD.	
5. The GP was informed of the recommendations in writing; an individualised clinical letter from the pharmacist based on a standard proforma. The letter detailed the recommendations and the rationale for the recommendations. This was followed up with a phone call.	

### Outcome measures

The primary outcome measure was the Neuropsychiatric Inventory-Nursing Home version (NPI-NH) at 3 months [14]. This is a caregiver administered questionnaire that assesses neuropsychiatric symptoms. Other outcomes included quality of life (EQ-5D/DEMQoL) [15, 16], cognition (sMMSE) [17], health economics (modified version of Client Services Receipt Inventory [CSRI]) [18] and prescribed medication (including implementation of the review; obtained from the care home medication record). Data were collected at 8 weeks, and 3 and 6 months (findings will be reported elsewhere).

An embedded process evaluation used individual semistructured qualitative interviews to explore the expectations and experiences of GPs and care home staff, including managers, both pre- and post-intervention. In addition, the chief investigator collected reflective comments from members of the team and participants (*n* = 9) to inform the potential design of the full trial. These accounts were collected during a short (up to 15 min) phone interview, which covered was the review helpful, and barriers and facilitators to participation in the study and implementation of the intervention.

All care home staff who received the behaviour change intervention were asked to complete two questionnaires. First, the Approaches to Dementia Questionnaire, which was administered pre-intervention, immediately after the training, and 3 months post-intervention; second, the Maslach Burnout Inventory – Human Services Survey which was administered pre- and 3 months post-intervention (findings will be reported elsewhere).

## Results

### Recruitment of care homes

Recruitment took far longer than expected. Recruiting six care homes was planned to take six months; it took 14 months to recruit five care homes. Despite two six-month extensions to the study period, it was not possible to recruit a planned sixth care home in the time available. Our original intention was to search the CQC web-site electronically, using the inclusion criteria, to identify eligible care homes. Due to the complexity of the database this did not prove possible, and it was necessary to look at each home individually on the website. Local authority and commercial websites (e.g. www.carehomes.co.uk) were searched using the same approach.

Our revised search strategy identified 82 eligible care homes. Three of these homes were recruited (conversion rate = 3.7%). Subsequently the support available from ENRICH was used; three ENRICH homes expressed an interest and one of these was recruited [19]. One care home was recruited by personal contacts – this home did not respond to the initial letter and follow-up.

At individual care home level, the decision regarding participation was largely driven by the care home manager, and personal contact between the research team and care home managers in following up initial invitations to participate was effective in securing care home sign up. The care homes recruited to the study were diverse (see Table 3 for further details).

**Table 3:** Characteristics of participating care homes

ID	Type	Organisation	Specialty	Number of beds
001	Nursing home	Local charity with small number (< 5) of care/nursing homes	Adults over 65	52
002	Residential home	Medium sized care home chain (50 to 100 care homes) across England	Adults over 65; Dementia	64
003	Nursing home	Small care home chain (< 5)	Adults over 65; Dementia	76
004	Nursing home	Single ownership	Adults over 65; Dementia	72
005	Nursing home	Single ownership	Adults over 65; Dementia	31 dementia (45 total)

### Time to recruit care homes

The time to recruitment for care homes was calculated according to the number of days between the initial approach from the research team to the care home manager, and the receipt of local ethical approval permitting recruitment of residents to begin in that care home. The mean number of days taken to recruit care homes was 236.6 (SD 127.2). This was partly because of the time taken to recruit the final two care homes: the care home recruited via ENRICH took 314 days to recruit, and the care home recruited via the personal contacts took 421 days. The first three care homes, which were recruited following an initial letter to the care home manager took a mean of 149 days (SD 31).

### Number/proportion of eligible residents in each care home and resident recruitment

Across the five participating care homes, 295 potential participants were available for eligibility screening (see Table 4). Of these, 108 (36.6%) met the inclusion criteria. The proportion of eligible residents varied from 29.2 to 58.1% across care homes.

**Table 4:** Eligible residents in participating care homes and recruitment rate

Care Home ID	Number eligible / number screened	Proportion eligible (%)	Number recruited	Proportion of eligible residents recruited (%)
001	17/52	32.7	10/17	58.8
002	26/64	40.6	10/26	38.5
003	24/76	31.6	6/24	25.0
004	21/72	29.2	5/21	23.8
005	18/31	58.1	3/18	16.7
ALL	108/295	36.6	34/108	31.5

Overall, 34 of the 108 residents were recruited to the study (conversion rate = 31.5%). Recruitment rates from individual care homes ranged from 16.7% (*n* = 3/18) in care home 005 to 58.8% (*n* = 10/17) in care home 001. Recruiting 34 individuals in total equates to a mean of 6.8 residents at each care home (range 3 to 10; Standard Deviation [SD] = 3.11). The number of individuals recruited to the study represented 75.6% of the original target (*n* = 45). Several additional potential participants were identified in the last care home recruited, but there was not enough time remaining in the study period to complete follow-up so these residents were not recruited.

### Time to recruitment for study participants

Time to recruit study participants was calculated from the number of days between ethical approval being granted for recruitment to begin in each care home, and the point at which the last participant at that care home was recruited to the study. Time to complete participant recruitment ranged from 117 to 349 days (mean = 219.6; SD = 84.2).

### Recruitment of GP practices

Consent from each participant’s GP was required for the medication review. Obtaining consent was straightforward in homes predominantly supported by a single general practice with strong links between the practice and the home (care homes 001, 003, 005). It was considerably more challenging in care homes supported by multiple practices (care home 002 supported by 4 practices and 004 by 2 practices).

In care home 002 it took over 3.5 months to obtain GP consent for every resident and required a strategic and time-consuming approach to primary care engagement. This approach included working closely with the Clinical Comissioning Group (CCG), Practice-based Pharmacists (PBP) and NIHR Clinical Research Network (CRN) primary care leads, a newsletter about the study specifically written for local GPs, an article in a local NIHR “Connect” Magazine, presentation at a GP Local CRN event on Dementia and attendance at a GP practice meeting. In care home 004, it was impossible to obtain GP consent for 2 recruited participants, for whom the personal consultees had assented, despite the earlier strategic approach.

### Retention rates of care homes and participants

**Fig. 1:**
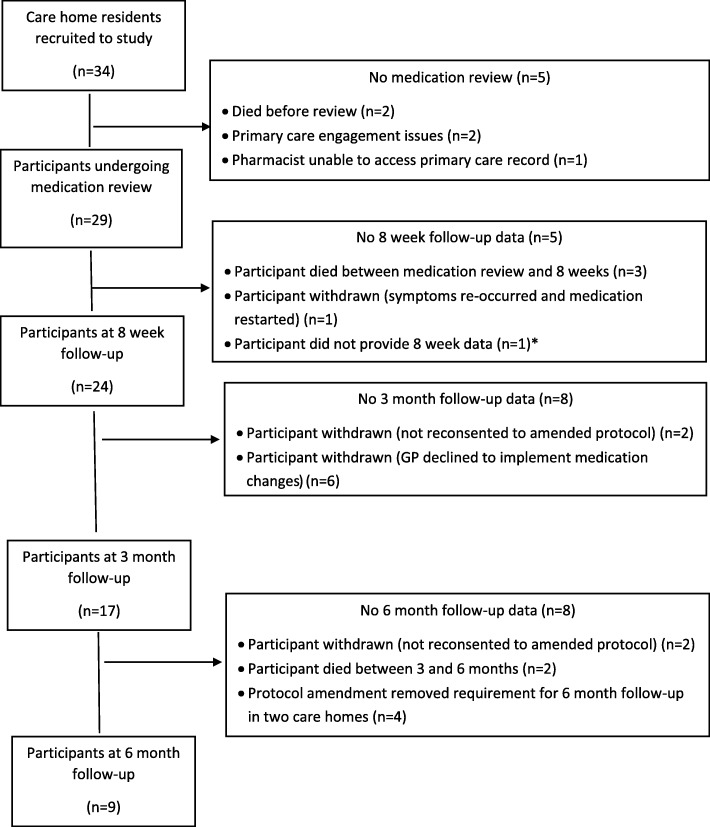
Participant retention through the study. Flow chart detailing participant retention throughout the study. * Participant who did not provide data at 8 weeks went on to provide both 3 and 6 month data

None of the five care homes withdrew from the study. Five study withdrawals (14.7%) occurred before the medication review could be undertaken. Further participants were withdrawn at 8 week (*n* = 5; NB: one of these 5 subsequently provided data at 3 and 6 months), 3 month (*n* = 8) and 6 month (*n* = 8) follow-up. Attrition rates by care home ranged from 67 to 83.3%. Figure 1 shows participant attrition and the reasons for loss to follow-up at each data collection point.

### Medication reviews: Recommendations and implementation

**Fig. 2:**
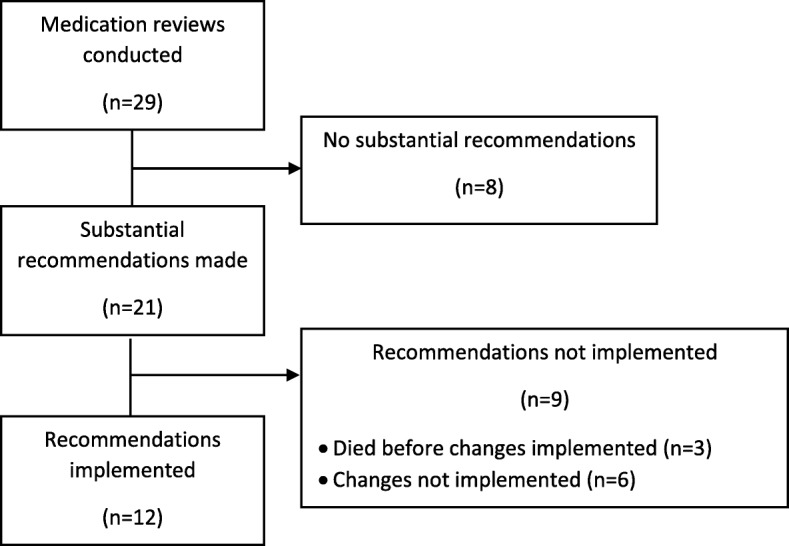
Consort diagram of medication review. Consort diagram detailing number of medication reviews and implementation

Medication reviews were conducted by specialist pharmacists for 29/34 study participants (85.3%) (Fig. 2). A written recommendation to stop or review medication was made for 21/29 participants (72.4%). The recommendations were implemented by the participants’ GP in 12 of the 21 medication reviews (57.1%). There was substantial variability in implementation of recommendations by care home, ranging from 0 to 100%. It took a mean of 98.4 days (range 33 to 138; SD = 42.5) to implement the recommendations. The protocol was amended so that the participant baseline assessment was repeated if the recommendation was not implemented within eight weeks of the medication review.

### Behavioural change intervention

One hundred and forty-two care staff attended the person-centred care workshop and received the Behavourial Change Intervention across the five homes (mean = 28.4; range 25 to 38; SD = 5.41). For further details of participation rates, see Table 5.

**Table 5:** Number of staff at each home participating in training and total number of staff

Care Home ID	Number of participating in training	Completion rate	Total number of staff in care home	Proportion of staff receiving training (%)
001	26	100%	72 (includes staff in non-caring roles)	36.1%^a^
002	25	100%	Approximately 50 carers	50%
003	26	100%	34	76.5%
004	27	100%	Approximately 63 staff in caring roles	42.9%
005	38	100%	Approximately 148 staff	25.7%^a^

^a^these figures include staff in a non-caring roles

In addition, 22 primary care clinical staff, including GPs (*n* = 14), GP trainees (*n* = 4), Practice Nurses (*n* = 3) and PBP (n = 1) across nine practices received the modified training primarily focussing on the treatment of BPSD. Four GPs were trained on the phone (one per training session); whereas 18 staff received training in five face-to-face sessions (mean of 3.6 per session).

### Qualitative evaluation of expectations and experiences of care staff

Face-to-face, semi-structured interviews were conducted [by NC] with 21 participants (Care Home Managers [CHM] = 5; GPs = 3; Care Staff [CS] = 13). This paper reports expectations and experiences of the feasibility study. Participants’ understanding of current guidance, person-centred care and details of changes in perceptions of people with dementia will be fully reported in a subsequent paper.

Participants found both the training and the medication review aspects of the feasibility study beneficial:

*“Very useful exercise...being provided the information it helps (to) make us sure that we’re prescribing appropriately.” (GP1, post-intervention).*

*“I did ask random staff after the training and they were all really positive, said they found it really interesting, really helpful and I’ve actually run it past my Learning and Development Partner who deals with my training in my area and they’ve actually sanctioned it being just as good as the (care home company) training.” (CHM2, post-intervention).*

Participants identified that after the training they were more likely to adopt a holistic approach with less reliance on medication:

*“I think staff now discuss and think slightly different from what they did before. Before it would be 'go to the nurse', it would be 'what meds can we give?' That’s still there with some but overwhelmingly it’s 'what can we do different?'” (CHM5, postintervention).*

*“I think there’s been very little (medication) put back. You know sometimes when you stop things, somebody immediately just worries about it and it just goes straight back on but I don’t think that’s happened.” (GP2, post-intervention).*

The practical training approach promoted adoption of patient-centred care, which underpinned this more holistic approach to care:

*“People don’t realise, a lot of time, their actions have a reaction and having– just examples and bringing it to the forefront of the mind, which is what the training does…they start thinking about things a little bit differently and I noticed that after the training the team did start actually trying to find what was working and what wasn’t working.” (CS13, post-intervention).*

The holistic approach, to the medication review, with the focus on quality and safety, rather than cost also promoted adoption of the intervention:

*“At the same time…a CCG pharmacist (was) going into the home to do sort of medication reviews…more of the point-of-view of reducing costs. Different approach from the MEDREV pharmacist…very much geared around using the evidence to increase the quality of care...doing it for…quality and safety rather than cost prioritisation.” (GP2, post-intervention).*

GPs identified that participation placed little burden on them, although some GPs identified barriers including time taken to implement the medication review:

*“The study was already set up to be as easy as it can be on GP time.” (GP1, post-intervention).*

*“That was just broadly because of timing and me not being able to…do it because of lots of things that happen in surgery.” (GP3, post-intervention).*

Overall, providing care staff with additional tools and skills to address behaviours that challenge appeared to have a positive impact on the attitudes and practices of care home staff:

*“The care home staff have been reassured by the training…sometimes when I’ve done medication reviews in the past they’ve been a little bit cautious. “Oh, I don’t think their family will like that”. “Oh, this person’s been on it for ages”…we’ve had a little bit of resistance and that’s gone a little bit.” (GP2, post-intervention).*

*“…the way that the training’s done it’s all about the VIPS and it’s making people really think…about the individual, what might be wrong with them, how to minimise those challenging behaviours…it’s giving people more tools to do their job better.” (CHM2, post-intervention).*

## Discussion

Increasing care home research is a key priority, and to the best of our knowledge this is the first study to report in detail the feasibility of a dual-purpose care home study involving staff training and medication review. Overall, recruitment was challenging and time-consuming, securing GP engagement was difficult, drop-out rates were high and, where substantial recommendations in relation to medication were made, these took a long time to implement, if implemented at all.

Like other care home research, recruitment was challenging [20-23]. The CQC database, as a search engine, had limited utility, confirming earlier findings (Personal Communication, Analisa Smythe, 18th October, 2017). It took 14 months rather than six months to recruit five care homes; and like other studies, the protocol was continually adapted as the team learnt from earlier experience [21]. This resulted in the need for five substantial ethical amendments during the study, which contributed to delays. Amendments included expanding the recruitment area, introducing the re-baseline procedure to account for the delay in implementation and in the final two care homes removing the six month follow-up (due to time limitations).

Our initial response rate of 3.7% is low compared to other studies that offered training to care homes; another study had a 10% response rate [21]. This was possibly because care home managers, the key decision makers, welcomed the offer of cost-free training, but were less likely to welcome a medication review, which could potentially lead to discontinuation of medication for behaviour that challenges. Informal feedback, obtained as reflective comments, from the managers and evidence from another study confirms this viewpoint; one observational/interview study on current practice for the treatment of behaviours that challenge had a similar response rate [20]. Like other studies we found that developing local relationships and using ENRICH were successful techniques [21].

It took a mean of 236.6 days to recruit homes. Like other researchers, recruiting GPs from multiple practices who provide care to participants living in a single care home was particularly challenging [22]. Our strategic approach to GP engagement was only partially successful and should be developed further for any larger trial.

At least 30% of residents were expected to meet the inclusion criteria with at least 18 potential participants per home and 7 to 8 recruited. The actual figures were 36.6% of residents with 21.6 potential participants per home and on average 6.8 recruited. On reflection, recruitment might have been improved by organising further meetings with relatives and greater involvement of care home staff in recruitment, although further training may be required for the care home staff.

Recent NICE guidance recommend that care providers should provide face-to-face training and mentoring to staff who deliver care and support to people living with dementia [24]. This training should include the management of behaviours that challenge including the appropriate use of medication [24]. MEDREV successfully developed and evaluated an acceptable and feasible training package, which was well received. A large number of staff (*n* = 164) received training about delivering person-centred care, and the use of psychotropics and reasons for reducing them. Furthermore, by combining staff training with a specialist medication review, the use of psychotropics was reduced [25].

The qualitative research and the reflective comments, obtained from GPs were very supportive of both the training and medication review. Staff were positive about both elements. The Behavioural Change Intervention appeared to train the care staff in person-centred care so that they would understand why reducing psychotropics is beneficial and support implementation of the recommendations from the medication review. The pharmacists who trained the GPs also reported good interaction particularly in face-to-face training, which encouraged greater participant engagement and reached more GPs.

Nearly 43% of the recommendations (*n* = 9/21) were not implemented. Other similar studies have found similar rates; for example one study found that 58.1% of recommendations by a clinical pharmacist were implemented [26]. The reasons for this, in our study, were not entirely clear although given the range in implementation rates between care homes, local context, and in particular the GP/care home/pharmacist relationship was likely an important factor. Informal feedback, obtained in the reflective comments, identified a perceived lack of integration with other secondary care medication reviews. The likelihood and speed of implementation may have increased with direct communication between the pharmacist and GP either by phone or face-to-face. Another possible avenue to explore is utilising the model of a practice-based pharmacist as a liaison between the specialist pharmacists conducting the medication reviews and the GP.

Other studies have found that GPs were broadly supportive of pharmacist medication reviews for BPSD and the implementation rate is similar to other studies involving clinicians implementing recommendations from a pharmacist [27, 28]. The relatively low uptake could be due to the additional time and effort needed to amend the prescription. Other studies suggest that GPs believe reducing anti-psychotic prescribing for BPSD could be achieved by increased availability of non-pharmacological approaches and staffing levels [28].

Even when supported, the medication review recommendations took on average 98.4 days to implement. This may have clinical and medico-legal implications. It also creates methodological problems for future studies: because it was impossible to know when the recommendation was likely to be implemented, collecting outcome data was challenging. One possible reason for the delay was the use of pre-prepared medication administration packs, which are prepared every month, for care homes. Care home staff also attributed the delay in implementation of recommendations to a general low priority for healthcare for older people; this needs further exploration in future research.

Whilst problems relating to medication optimisation in care homes and in people living with dementia are widely acknowledged, there is very little research on interventions to optimise medication in care home residents [29-31]. One trial has investigated a PCT/CCG-led medication review [32]. The homes in our study already received regular medication reviews from CCG pharmacists; suggesting that CCG pharmacists may lack expertise to review psychotropics. Furthermore, the GPs, in our study, appreciated the clinical and quality focus of the medication reviews.

### Limitations

This study was conducted in a single region in the UK and had a limited number of participants. However, we recruited and retained a range of care homes with differing characteristics (type of home, sociodemographic characteristics of local area). The original aim was to recruit a representative sample of staff from each home. However, only three members of staff were recruited from the final two homes, due to the difficulty and subsequent delay in recruiting these homes. Only three GPs were recruited despite efforts to recruit more. No pharmacists were interviewed; however the feasibility issues in relation to the medication review were captured in the interviews with care staff and GPs, and the reflective comments received.

### Policy implications

Healthcare policy must continue to focus on optimising medication usage in care homes, including the appropriate treatment of BPSD. MEDREV developed an acceptable and feasible training programme which included the appropriate use of medication, in line with NICE guidance, suggesting that this may be a promising policy approach [24]. Since this study, NHS England has invested in pharmacy to support medication optimisation in care home [33]. This research suggests, that to successfully optimise medication, these pharmacy staff need to develop robust ways of working across organisational boundaries linking primary, secondary and social care.

There are also implications for research policy makers. Recruiting the care homes and people with living dementia was time-consuming and difficult, confirming other studies. The NIHR and other funders have prioritised high quality research both in care homes, and on medication optimisation in older people [31, 34]. Yet despite this, there is limited research on medication optimisation in care homes [35] perhaps because care home research involving medication optimisation is uniquely challenging, as we found. This suggests that research into medication optimisation in care homes needs to be a specific priority.

### Future research

One of the key challenges in this study was the delay in implementation of recommendations. Whilst our solution, of repeating the baseline measurements, might work for a single location feasibility study, when the chief investigator is able to work closely with the Clinical Study Officers, it is less likely to work for a larger multiple centre study. Expert recommendations on medication optimisation did not appear to be implemented in a significant minority of residents; this needs further investigation. From the care home point of view, it may be a question of who has the greater authority, the GP or the pharmacist and established relationships.

GP engagement could be improved by holding an initial event very early in the study. This event should carry Continuing Professional Development accreditation from the appropriate Royal College and include education from expert speakers, ideally with international reputations, in addition to information on the study.

Specialist pharmacists may not have had time to build a good relationship with the GP and without good communication and trust implementation of the recommendations may be challenging as we found. Since this study began, there has been significant investment in primary care clinical pharmacy including within care homes [36-38]. These practice-based pharmacists (PBP) are perhaps ideally placed to deliver the medication review; they have access to records and the autonomy to change the repeat template particularly if an independent prescriber. Involving such PBPs in the delivery of the medication review could address some of the feasibility issues identified and is a hypothesis for a future trial. Yet this area may be outside the scope of their practice and competency. Future research should explore the best way for pharmacy staff to deliver this specialist medication review and the training requirements.

## Conclusion

The feasibility study contained two linked elements; staff training and medication review. We found it feasible to develop, deliver and evaluate a well-received staff training programme both in the care home and the GP surgery. The dual intervention appeared to increase the ability of care staff to manage BPSD appropriately with less reliance on medication. Although we found a clear need for specialist medication review of psychotropics for care home residents with dementia, the medication review would require significant modification for full trial.

## Abbreviations

BPSD: Behavioural and Psychological Symptoms of Dementia
CHM: Care Home Manager
CRN: Clinical Research Network, an NIHR Research Network Coordinating Centre for UK clinical research studies in the NHS
CS: Care Staff
ENRICH: Enabling Research in Care Homes an NIHR research ready initiative for care homes in the UK
GP: General Practitioner
MRC: Medical Research Council
PBP: Practice Based Pharmacist
SD: Standard Deviation
VIPS: Valuing personhood; Individual needs; Personal perspectives; Social environment
